# Opthalmological Wards

**Published:** 1871-12

**Authors:** 


### OPTHALMOLOGICAL WARDS.

---

### Service of PROF. JONES.

---

CASE I. SYPHILITIC IRITIS.—This is a case of unusually severe syphilitic iritis; the eye bulges forward considerably and the chances are that the iris will be found attached to the lens, in which case an operation must be performed upon the iris. The conjunctiva and sclerotica are very red, but the front of the ball is of a whitish yellow color from the presence of pus in the anterior chamber; the aquæous humor has become purulent.

At this point the patient, while being put under the influence of ether, exhibited a difficulty in breathing, and the lecturer directed the tongue to be pulled forward; this maneuvre gives immediate relief from the slight obstruction to respiration and is more convenient than turning the patient upon his side.

The evacuation of the pus often gives great relief to the eye; as the aquæous humor is secreted rapidly and refills the chamber a couple of hours after its evacuation, the chamber may be tapped every day if necessary. A speculum is introduced to keep the lids apart, and an incision is made into the cornea, through which the purulent fluid is emptied. The iris proves not to be attached to the lens and is not interfered with; the operation is confined to the simple tapping of the anterior chamber. A dry compress is then put upon the eye, and in 36 hours union will be completed without any danger.

---

Case II. In a second case of iritis which is exhibited, the cornea is comparatively clear; there is no muco-purulent discharge and less lachrymal discharge than usual in this affection there is a slight exudation on the iris, making it brownish while the other iris is blue. In the treatment, the pupil will be dilated at once with atrophia in order to remove the iris from proximity to the lens and possible attachment; Dover's powder gr. V will be given and a mustard foot bath used at night.

---

Case III. Conjunctivitis.—A patient who was examined by the class about a month ago is again exhibited in order to show the effects of the treatment used upon him for conjunctivitis. The membrane is much less inflamed, still red but not rough, and the ulceration which had existed is gone. Besides the local application of sulphate of copper and zinc, the syrup of iodide of iron was administered.

Another patient with conjunctivitis is a child a year and a half old, who was found in the streets after the great fire and was brought to the Hospital, where he will probably be adopted by one of the Sisters of Mercy. When first seen he could scarcely open the left eye and some of the lashes were misdirected. The offending hairs were removed and citrine ointment 1 part to cream 8 parts, with some astringent, was applied, and gave relief to the inflammation.

